# People would rather see a physician than a dentist when experiencing a long-standing oral ulceration. A population-based study in Spain

**DOI:** 10.4317/medoral.23292

**Published:** 2020-05-10

**Authors:** Pablo Varela-Centelles, Juan Seoane, Yaima Ulloa-Morales, Ana Estany-Gestal, Andrés Blanco-Hortas, María J. García-Pola, Juan M. Seoane-Romero

**Affiliations:** 1 Praza do Ferrol Health Centre, EOXI Lugo, Cervo e Monforte, Galician Health Service, Spain; 2 Department of Surgery and Medical-Surgical Specialities, School of Medicine and Dentistry, University of Santiago de Compostela, Spain; 3 Unit of Methodology of the Research, Health Research Institute of Santiago de Compostela, Spain. Lugo University Hospital, Spain; 4 Department of Surgery and Medical-Surgical Specialities, School of Medicine and Health Sciences, University of Oviedo, Spain

## Abstract

**Background:**

Primary care physicians have been reported to be the first choice for patients with oral ulcerations. This study investigates the health-seeking behaviour of lay public in Galicia (North-western Spain) if experiencing a long-standing oral ulceration.

**Material and Methods:**

Cross-sectional population-based survey of randomly selected respondents conducted from March 1, 2015 to 30 June 2016.

**Results:**

A total of 5,727 pedestrians entered the study (response rate: 53%), mostly in the 45-64 age group (30.2%; n=1,728), 47.7% of them (n=2,729) were males. Most participants (42.1%; n=2,411) reported to visit their dentist once a year and had secondary or compulsory education as their highest educational achievement (28.18%, n=1,614; 28%, n=1,600 respectively). When questioned what they would do if they had a wound/ulceration lasting longer than 3 weeks, most participants answered they would go to see their primary care physician (62.8%; n=3,597) and less than one quarter of the sample (23.8%; n=1,371) would seek consultation with their dentist.

**Conclusions:**

General Galician population would seek professional consultation about a long-standing oral ulceration, relying mostly on primary care physicians. Those neglecting these lesions are elderly, less-schooled people and unaware of oral cancer.

** Key words:**Oral ulceration, oral cancer, patient attitudes, surveys and questionnaires, Spain

## Introduction

Oral cancer constitutes a public health problem for most countries with an average 5-year survival of 50-60%. A large proportion of patients (about 50%) had been diagnosed at late stages during the last four decades. In addition, lip, oral cavity, and pharyngeal cancers incidence is increasing worldwide and estimations point at 856,000 new cases by 2035 (1).

It has been suggested that early diagnosis is the most important prognostic factor for overall survival (2). Thus, long time intervals to oral cancer diagnosis seem to influence both advanced TNM-stage at diagnosis (2-fold risk) and survival to this tumour. Particularly, the patient and the primary care intervals are the longest time-periods in the path to diagnosis and they have proved to be a risk factor for advanced stage at diagnosis (3) and mortality from oral cancer (4). In this vein, detection of bodily changes and perception of reasons to discuss symptoms with a primary healthcare professional are paramount and define the appraisal and help-seeking intervals by the patient (5). Therefore, approaches to improve survival rates have to focus on the patient interval, and disclosing patients’ attitudes when noticing the most frequently reported first oral cancer sign -an unexplained oral ulceration standing longer than three weeks- (6) seems to be a logical basis for any educational intervention on this issue.

Thus, the aim of this study was to investigate the health-seeking behaviour of lay people in Galicia (North-western Spain) if experiencing a long-standing oral ulceration.

## Material and Methods

A cross-sectional, population-based study was designed using a questionnaire applied face-to-face to randomly selected members of the public in Galicia (North-western Spain) from 1 March 2015 to 30 June 2016, by 14 specifically trained interviewers (postgraduate (n=7) and undergraduate dental students (n=2), 1 undergraduate medical student, 2 nurses, and 2 nurse assistants).

The instrument used in the study was a modification or the questionnaire originally developed by Rogers *et al* (7) in English language. The original instrument was translated into both Spanish and Galician and then back into English (double translation). Some items in the instrument (employment, academic achievements, and registration with a dentist) were also modified to adapt them to the Galician sociocultural environment. The resulting instrument was piloted in a group of clinicians in a first instance and, after reformulations and corrections, was piloted again in a group of undergraduate dental students and senior volunteers at a community centre.

Sample size was determined by quota sampling considering an accessible population of 5% and an expected percentage of response of 28% (7). The resulting sample size of 10,804 people permitted a power of 0.8% for estimating the proportion of oral cancer aware people, presuming a value of 25%.

Only pedestrians over 18 entered the study. The exclusion criteria were: being younger than 18, mentally disabled, or poor command of any of the official languages of the region (Galician or Spanish).

Galicia is an autonomous region with 2,708,339 inhabitants unevenly distributed in 29,574.4 Km2, with a yearly gross domestic product per capita of 21,358 € and a life expectancy at birth of 82.78 years. The region is served by a public, free, universal health service, characterised by a strong and accessible primary care level.

Data were obtained in all four capitals of the Galician provinces at four different commercial and administrative areas in each city on different days and times, in a kind of pathfinder survey method (8).

The interviewers participated in a 1 hour-long workshop which included discussion of the items in the instrument and their related ethical aspects, together with a role-playing session and a series of interviews to volunteer subjects (undergraduate dental students) under the supervision of a psychologist.

Each questionnaire was numbered, which permitted an assessment of the data coding and mechanization process, before transferring them to the R v3.3.2, MASS, and nnet statistical packages for analyses.

The results of the descriptive analysis are presented as frequencies and percentages. Bivariate analysis was undertaken using the Chi Square/Fisher’s exact test. A logistic regression analysis to disclose the features of those choosing between a primary care physician and a dentist was also performed. The significance level chosen for all test was 5%.

The study protocol was approved by the Santiago-Lugo Committee for Ethics in Research (number 2014/600). This investigation complied with the Spanish regulations and the Helsinki Declaration on ethical principles for medical research involving human subjects. The results are presented according to the STROBE guidelines (Strengthening the Reporting of Observational studies in Epidemiology) (9).

## Results

A total of 5,727 pedestrians entered the study (response rate: 53%), mostly in the 45-64 age group (30.2%; n=1,728), 47.7% of them (n=2,729) were males. Most participants (42.1%; n=2,411) reported to visit their dentist once a year and had secondary or compulsory education as their highest educational achievement (28.18%, n=1,614; 28%, n=1,600 respectively).

When questioned what they would do if they had a wound/ulceration lasting longer than 3 weeks, most participants answered they would go to see their primary care physician (62.8%; n=3,597) and less than one quarter of the sample (23.8%; n=1,371) would seek consultation with their dentist. Self-treatment (1.8%) is the reported behaviour predominant among those circulating an alternative path (11.5%) to diagnosis/treatment (Table 1).

**Table 1:**
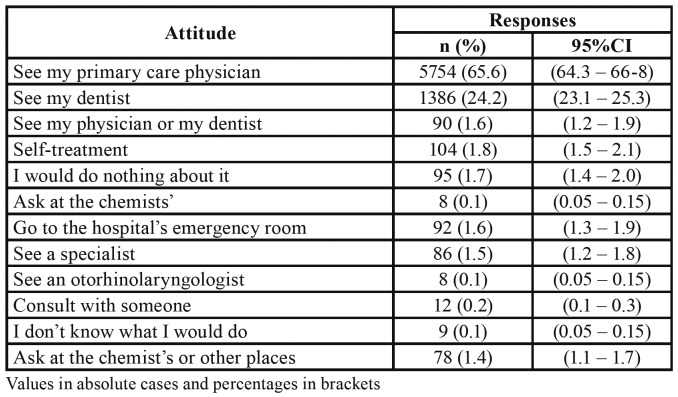
Attitudes towards a non-healing ulceration after three weeks (n=5727).

The distribution of these attitudes according to the socio-demographic variables considered in the study is summarised in Table 2. This Table shows males predominate among those who would ask a physician (67.2%), whereas females preponderate in the group choosing to visit a dentist (26.8%).

**Table 2:**
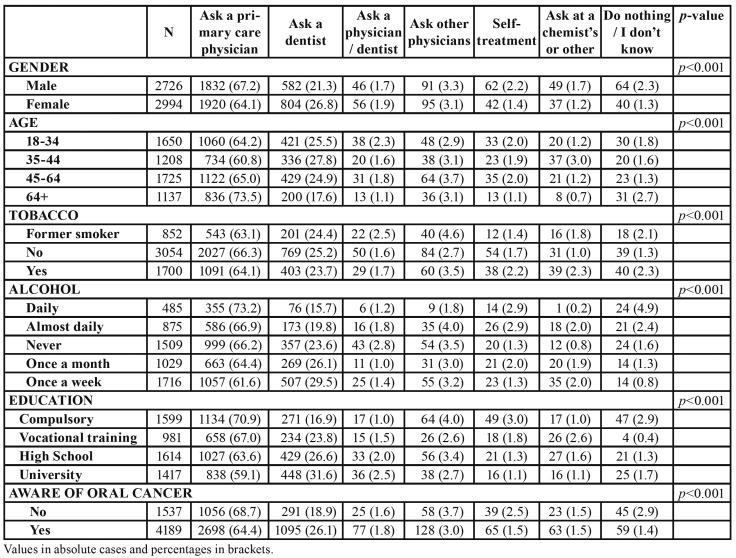
Distribution of attitudes towards a long-standing oral ulceration.

The same phenomenon occurs for the elder and younger groups of participants and for the lowest and highest educated volunteers in the sample: elder (73.5%) and less educated (70.9%) interviewees would rather see a physician in case of a long-standing oral ulceration.

**Figure 1:**
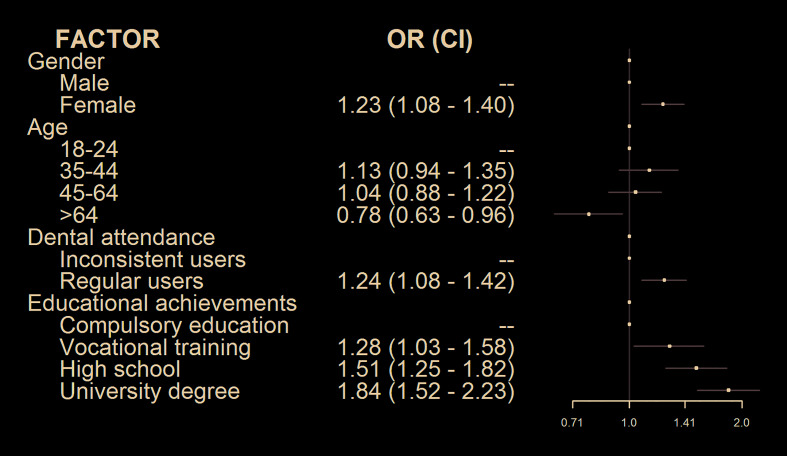
Logistic regression analysis of help-seeking attitudes (primary care physician vs. dentist).

Females have more chances to go to a dentist when experiencing a long-lasting oral ulceration (OR= 1.23; 95% CI: 1.08 – 1.40), as occurs with participants regularly using dental services (OR= 1.24; 95% CI: 1.08 – 1.42). Chances to go to a dentist also increase with the participants’ educational level (Fig. 1).

Knowledge about the existence of oral cancer also seems to influence the reported behaviour towards a long-standing mouth ulceration: people reporting no knowledge on oral cancer would choose to visit a physician (68.7% vs. 31.3%) and seem to be more prone to stoic or risky behaviours (Table 2).

## Discussion

- Strengths and weaknesses of the study

Our quota-sampling approach resulted in an equiTable balance of age and gender in the sample, and face-to-face interviews permitted a better feeling for people’s responses than would be possible through mail (7) or by telephone interview, this latter is also limited by the growing number of homes using only mobile phones (10). To the best of our knowledge, this is the largest population-based study on this topic with a high participation rate. In addition, the method for volunteer recruitment (at the busiest commercial and administrative areas in the region during several months at different times), combined with knowledgeable, specifically trained interviewers, may have well contributed to increase the external validity of the study. However, this kind of studies always depend on self-reported data and some variations have to be expected regarding actual attitudes. In this particular situation -where there is no “right” answer- and the reported responses are consistent with data from actual cancer patients (11), this limitation is highly unlikely to have conditioned our results.

Our research might have been affected by a hypothetical selection bias, where pedestrians with lower health literacy may have refused to participate in the survey more frequently than other people. As health literacy is somehow related to educational achievements (12) and most participants in the sample where in the compulsory education group in a proportion similar to their weight in the general population of the region. This hypothetical bias, if existed, may have had a minor influence on our results.

Considering the issues discussed above and the large size of the sample in a region where their capital cities are well communicated with their metropolitan areas, we understand our results offer reliable data on this topic, which may well be extrapolated to elsewhere in Spain.

- Justification for the research model

Recognition of a symptom as a potential danger is a challenge for patients, and the absence of pathognomic oral cancer signs and symptoms could explain long diagnostic delays attributed to the patient. Symptoms persistence seems to be paramount in the patient’s decision-making processes of seeking help (13). In this sense, an unexplained ulceration in the oral cavity >3 weeks is red-flag symptom in the new NICE head and neck cancer guidelines, with a higher positive predictive value than the red or red and white patches for oral cancer diagnosis (6).

Besides, oral ulcerations represent the most frequent clinical sign of oral cancer, and this subtype is usually (up to 60%) diagnosed at later stages with implications in poor survival, although available evidence remains equivocal (14).

- Reported attitudes towards a non-healing ulceration

Reports on the prevalence of oral ulcerations in the general adult population in Southern Europe have described frequencies somewhere between 2.5%-10% (15,16), mostly due to local trauma, iatrogenia, aphthae, infections, haematological disorders, malabsorption states, cutaneous diseases, or connective diseases (17). This relatively high prevalence of oral ulcerations, and the large proportion of people who had ever experienced one (18) may anticipate knowledge of the natural history of a typical oral ulcer and could explicate the high proportion (> 88%) of participants who would consult with a primary care professional about a long-standing one.

On the other hand, stoicism, self-medication or erratic navigation through the healthcare system, are attitudes some participants (11.5%) would take probably due to a reinterpretation of symptoms (signs) as minor conditions, which could cause a delay in the diagnosis of a potential neoplasm (5,13).

Primary care physicians consistently are the first choice for patients with oral ulcerations both in our study and in the literature (7), only behind traditional remedies in certain countries, which have been proved to increase the risk for presenting with advanced disease stage at diagnosis (19). Studies on cancer patients confirm the preference for physicians (20), with the only exception of Japan, where dentists are reported to be the clinician of choice (21).

This physician preference is particularly marked in our study for those males, < 64, unaware of oral cancer, and with compulsory education as their highest educational achievement. Almost a third of university graduates would choose a dentist in a first instance. This may well represent a spurious relationship linked to an association of the variables education and income and to the very little oral healthcare treatments for adults provided by the Spanish National Health System: the subgroup of younger, highly educated people would visit a dentist more frequently than their fellow participants as most dental treatments are provided on a private basis (22).

The preference for physicians over dentists when experiencing an oral mucosal problem raises concern on aspects such as the concept population has about dentists’ competence on issues “beyond the tooth territory”.

- Physician vs. dentist

Professional (primary care) diagnostic delay is strongly related to tumour stage at the time of diagnosis (3). Despite the aforementioned patient preference for physicians, information on their competence for early oral cancer diagnosis is scarce (23). Some reports have hypothesized about a relationship between diagnostic delay and the qualifications of the clinicians particularly among dentists and physicians with equivocal results (22). However, some studies reporting on general medical practitioners’ awareness of risk factors and clinical appearance of oral cancer state their performance is poorer than that of dentists (24).

- Clinical implications and recommendations

Self-medication, either by over-the-counter formulations or traditional remedies, have been reported to increase diagnostic delay, as well as the participation of off-clinical counsellors (25), who should also be considered in any oral cancer-related educational intervention. In this vein, oral cancer patients have indicated the potential usefulness of drastic visual aids on posters and leaflets in dental and general medical practitioners’ offices and pharmacies (26). Previous reports have described a high oral cancer diagnostic ability for Spanish dentists (27) but no information is available for physicians on this topic. In this sense, studies on the competence of Spanish general medical practitioners in diagnosing oral cancer are needed in view of our results, as well as potential educational interventions targeted to these professionals. Besides, barriers to dental care for patients experiencing red-flag symptoms and signs should be identified and removed.

Oral cancer does not seem a frequent topic on health promotion activities (28) and oral cancer survivors find that lay public should be encouraged to undertake regular medical and dental check-ups and to seek advice on oral symptoms as soon they have even the slightest concern.

## Conclusions

General Galician population would seek professional consultation about a long-standing oral ulceration, relying mostly on primary care physicians. Those neglecting these lesions are elderly, less-schooled people and unaware of oral cancer.

## References

[1] Shield KD, Ferlay J, Jemal A, Sankaranarayanan R, Chaturvedi AK, Bray F (2017). The global incidence of lip, oral cavity, and pharyngeal cancers by subsite in 2012. CA Cancer J Clin.

[2] Ghantous Y, Yaffi V, Abu-Elnaaj I (2015). Oral cavity cancer: epidemiology and early diagnosis. Refuat Hapeh Vehashinayim.

[3] Gómez I, Seoane J, Varela-Centelles P, Diz P, Takkouche B (2009). Is diagnostic delay related to advanced-stage oral cancer? A meta-analysis. J Oral Sci.

[4] Seoane J, Takkouche B, Varela-Centelles P, Tomás I, Seoane-Romero JM (2012). Impact of delay in diagnosis on survival to head and neck carcinomas: a systematic review with meta-analysis. Clin Otolaryngol.

[5] Weller D, Vedsted P, Rubin G, Walter FM, Emery J, Scott S (2012). The Aarhus statement: improving design and reporting of studies on early cancer diagnosis. Br J Cancer.

[6] Tikka T, Pracy P, Paleri V (2016). Refining the head and neck cancer referral guidelines: a two-centre analysis of 4715 referrals. Br J Oral Maxillofac Surg.

[7] Rogers SN, Hunter R, Lowe D (2011). Awareness of oral cancer in the Mersey region. Br J Oral Maxillofac Surg.

[8] Husain FA, Tatengkeng F (2017). Oral Health-Related Quality of Life Appraised by OHIP-14 Between Urban and Rural Areas in Kutai Kartanegara Regency, Indonesia: Pilot Pathfinder Survey. Open Dent J.

[9] Vandenbroucke JP, Von Elm E, Altman DG, Gøtzsche PC, Mulrow CD, Pocock SJ (2009). Strengthening the reporting of observational studies in epidemiology (STROBE): explanation and elaboration. Gac Sanit.

[10] Hertrampf K, Wenz HJ, Koller M, Wiltfang J (2012). Public awareness about prevention and early detection of oral cancer: A population-based study in Northern Germany. J Craniomaxillofac Surg.

[11] Santos LC, Batista Ode M, Cangussu MC (2010). Characterization of oral cancer diagnostic delay in the state of Alagoas. Braz J Otorhinolaryngol.

[12] Protheroe J, Whittle R, Bartlam B, Estacio EV, Clark L, Kurth J (2017). Health literacy, associated lifestyle and demographic factors in adult population of an English city: a cross-sectional survey. Health Expect.

[13] Scott SE, Grunfeld EA, Auyeung V, McGurk M (2009). Barriers and triggers to seeking help for potentially malignant oral symptoms: implications for interventions. J Public Health Dent.

[14] Seoane-Romero JM, Vázquez-Mahía I, Seoane J, Varela-Centelles P, Tomás I, López-Cedrún JL (2012). Factors related to late stage diagnosis of oral squamous cell carcinoma. Med Oral Patol Oral Cir Bucal.

[15] Pentenero M, Broccoletti R, Carbone M, Conrotto D, Gandolfo S (2008). The prevalence of oral mucosal lesions in adults from the Turin area. Oral Dis.

[16] García-Pola Vallejo MJ, Martínez Díaz-Canel AI, García Martín JM, González García M (2002). Risk factors for oral soft tissue lesions in an adult Spanish population. Community Dent Oral Epidemiol.

[17] Scully C, Shotts R (2000). ABC of oral health. Mouth ulcers and other causes of orofacial soreness and pain. BMJ.

[18] Gill Y, Scully C (2007). Mouth ulcers: a study of where members of the general public might seek advice. Br Dent J.

[19] Kerdpon D, Sriplung H (2001). Factors related to advanced stage oral squamous cell carcinoma in southern Thailand. Oral Oncol.

[20] Grafton-Clarke C, Chen KW, Wilcock J (2019). Diagnosis and referral delays in primary care for oral squamous cell cancer: a systematic review. Br J Gen Pract.

[21] Onizawa K, Nishihara K, Yamagata K, Yusa H, Yanagawa T, Yoshida H (2003). Factors associated with diagnostic delay of oral squamous cell carcinoma. Oral Oncol.

[22] Bravo M, San Martín L, Casals E, Eaton KA, Widström E (2015). The healthcare system and the provision of oral healthcare in the European Union member states. Part 2: Spain. Br Dent J.

[23] Gómez I, Warnakulasuriya S, Varela-Centelles PI, López-Jornet P, Suárez-Cunqueiro M, Diz-Dios P (2010). Is early diagnosis of oral cancer a feasible objective? Who is to blame for diagnostic delay?. Oral Dis.

[24] Carter LM, Ogden GR (2007). Oral cancer awareness of general medical and general dental practitioners. Br Dent J.

[25] Varela-Centelles P, Pedrosa R, Lopez-Niño J, Sánchez M, Gonzalez-Mosquera A, Mendez A (2012). Oral cancer awareness at chemist's and herbalist's shops: new targets for educational interventions to prevent diagnostic delay. Oral Oncol.

[26] Rogers SN, Vedpathak SV, Lowe D (2011). Reasons for delayed presentation in oral and oropharyngeal cancer: the patients' perspective. Br J Oral Maxillofac Surg.

[27] Seoane-Lestón J, Velo-Noya J, Warnakulasuriya S, Varela-Centelles P, Gonzalez-Mosquera A, Villa-Vigil MA (2010). Knowledge of oral cancer and preventive attitudes of Spanish dentists. Primary effects of a pilot educational intervention. Med Oral Patol Oral Cir Bucal.

[28] Shimpi N, Jethwani M, Bharatkumar A, Chyou PH, Glurich I, Acharya A (2018). Patient awareness/knowledge towards oral cancer: a cross-sectional survey. BMC Oral Health.

